# Translucency of Lithium-Based Silicate Glass–Ceramics Blocks for CAD/CAM Procedures: A Narrative Review

**DOI:** 10.3390/ma16196441

**Published:** 2023-09-27

**Authors:** Alessandro Vichi, Zejiao Zhao, Mahdi Mutahar, Gaetano Paolone, Chris Louca

**Affiliations:** 1 Dental Academy, University of Portsmouth, Portsmouth PO1 2QG, UKchris.louca@port.ac.uk (C.L.); 2 Dental School, IRCCS San Raffaele Hospital, Vita-Salute University, 20132 Milan, Italy

**Keywords:** lithium disilicate, lithium silicate, translucency, contrast ratio, translucency parameter

## Abstract

Amid chairside CAD/CAM materials, the use of lithium-based silicate glass–ceramics (LSGC) has been steadily increasing. This review aims to report on the translucency of these materials and the variables used to measure it. An electronic search was performed within the PubMed database within the period between 2 June 2011 and 11 September 2022. English-language papers investigating the translucency of IPS e.max CAD, Celtra Duo, Suprinity PC, Initial LiSi Block, Amber Mill, N!ce, and CEREC Tessera LSGC CAD/blocks were included in the search strategy. After an initial retrieval of 160 papers, the application of exclusion criteria, and the screening of abstracts and then of full texts, 33 papers were included in the study. The retrieved materials, with different degrees of translucency (LT, HT), were IPS e.max CAD (n = 33), Suprinity PC (n = 8), and Celtra Duo (n = 1). Concerning the examined colors, the most used was A2 (n = 20), followed by A1 (n = 8) and A3 (n = 2). The translucency parameter (TP) was the most used method (n = 30) with respect to the contrast ratio (CR) (n = 11) to assess translucency. Five papers measured both. Several specimens’ thicknesses (0.5–4 mm) were investigated, with 1 mm (n = 23) being the most frequently analyzed. While a general tendency could be identified, conflicting results among different papers were reported.

## 1. Introduction

Nowadays, the demand for optimum aesthetics has become a primary criterion for successful restorative treatments. This requires that the physical and optical properties of restorative materials match those of natural dentition [1,2]. Recently, a huge variety of metal-free materials have been introduced to both laboratory and chairside CAD/CAM technology [3,4,5,6,7,8,9,10].

The selection criteria can be challenging and are often based on mechanical and optical (aesthetic) characteristics. Generally speaking, there is an inverse relationship between these two properties. Materials with higher crystalline content are characterized by higher mechanical properties but higher opacity, while a higher glass content determines higher translucency but is characterized by lower mechanical performance [11]. The great advantage of metal-free materials with respect to porcelain fused to metal is that they can be penetrated by light, thus emulating the optical characteristics of natural teeth [12]. Correct material selection requires also that the optical properties of the underlying core be taken into consideration [13]. While an opaque material can be useful to mask a discolored background, translucent material is more indicated for a naturally colored substrate [14]. Understanding translucency is critical in the selection of materials and is of high clinical significance.

Among chairside CAD/CAM materials, lithium-based silicate glass–ceramics (LSGC) use has been increasing over the last few years [15]. LSGCs are composed of Li_2_O as the main oxide next to SiO_2_. According to the prevalent phase in which materials are crystallized, a classification has been proposed: “lithium disilicate” for the ones mainly composed by an Li_2_Si_2_O_5_ phase, “lithium silicate” for the ones crystallizing mainly in the Li_2_SiO_3_ phase, and “lithium (di)silicate” for materials composed by significant fractions of both phases [16].

In analyzing the components of the CAD/CAM systems available on the dental market, the following LSGCs have been identified: IPS e.max CAD (Ivoclar Vivadent, Schaan, Liechtenstein), Suprinity PC (VITA ZahnFabrik, Bad Sackingen, Germany), Celtra Duo (Dentsply Sirona, Charlotte, NC, USA), GC Initial LiSI Block (GC, Tokyo, Japan), Amber Mill (Hass, Gangwon-do, Korea), Tessera (Dentsply Sirona, Charlotte, NC, USA), and N!ce (Straumann, Basel, Switzerland). The materials and their chemical nature, retrieved from manufacturers’ technical datasheets, are listed in Table 1.

Some of the CAD/CAM LSGCs (IPS e.max, Suprinity PC, Tessera, and Amber Mill), to achieve final mechanical and optical properties, require a thermal treatment (crystallization) that needs to be performed in a dental furnace [17], while others (Celtra Duo, N!ce, and GC Initial LiSI CAD) do not require thermal treatment, as they are crystallized by the manufacturers [10].

CAD/CAM LSGC blocks are generally available in various shades and translucencies, usually low translucency (LT) or high translucency (HT). The translucency of aesthetic materials can be measured in different ways. The two most common methods are the contrast ratio (CR) and the translucency parameter (TP) [18,19,20]. While information is available in the scientific literature on the mechanical properties of CAD/CAM LSGC, the optical properties are less investigated, particularly regarding translucency. Therefore, the objective of the present review is to identify and discuss the different studies that have evaluated the translucency of CAD/CAM LSGC restorative materials.

## 2. Materials and Methods

### 2.1. Search Strategy

An electronic search of the literature was performed on PubMed on 11 September 2022. The survey covered the period from 2 June 2011 to 11 September 2022.

As this literature review was mainly aimed at providing a reference for practical interest to the dental community, it was decided to formulate the query using commercial names. The search strategy was the following: ((e-max) OR (Celtra) OR (N!ce) OR (LiSi CAD) OR (Suprinity) OR (GC Initial) OR (Amber Mill) OR (Tessera)) AND (translucency).

### 2.2. Inclusion Criteria

Full-text, English-language publications analyzing the translucency of CAD/CAM LSGC were included.

### 2.3. Exclusion Criteria

Publications were excluded if the assessment of translucency was not performed following an instrumental CR (contrast ratio) or a TP (translucency parameter) assessment. Therefore, studies based on a visual assessment of translucency were excluded. Also, as the study addressed milled versions of LSGC, studies analyzing translucency on pressed versions of LSGC were excluded.

### 2.4. Data Extraction

A data collection form was created and used to assess the experimental variables to measure specimens’ translucency. Six variables were assessed for every paper: (1) material type, (2) material shade, (3) material translucency, (4) specimen thickness, (5) the method used for translucency measurement (CR or TP), and (6) translucency value. The variables were recorded and tabulated in Excel sheets. Studies in which data on a certain variable were lacking or could not be calculated were entered as “not reported” (n.r.) for the variable in question. The selected articles are included in the reference list. A further search of the reference lists of the selected articles was also performed to eventually identify other relevant papers.

## 3. Results

**Figure 1:**
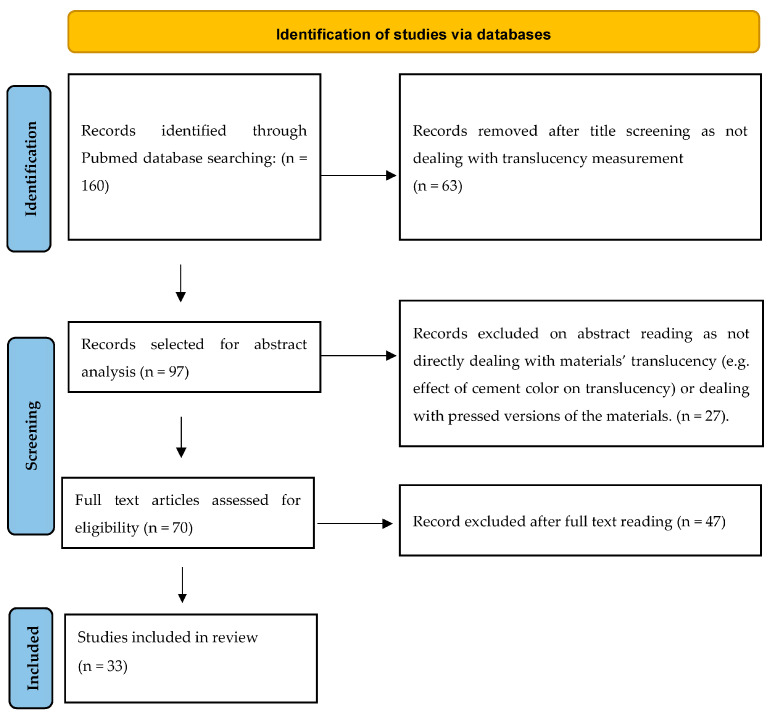
Stages of the study selection process.

The electronic search retrieved 160 titles for possible inclusion in the review. After initial elimination based on the titles, 97 abstracts were retrieved. For the selection of studies, two authors (A.V. and Z.Z.) independently reviewed the titles of the studies, according to the inclusion criteria for abstract reading. The Kappa score for interexaminer agreement on the screened abstracts was 0.89. After abstract screening, the full text of 70 articles was obtained. A further search of the reference lists of the selected articles did not result in additional articles being added. After full-text reading, 33 studies were found to be qualified for inclusion in the review (Figure 1). Among the retrieved papers, three LSGC-based materials were analyzed for translucency: IPS e.max CAD (n = 33), Suprinity PC (n = 8), and Celtra Duo. LSGCs with more than one translucency degree were also investigated. Both translucencies (HT and LT) were equally investigated (n = 18). A few papers (5 out of 33) did not mention the material’s translucency degree. The most investigated material shade was A2 (20), followed by A1 (8) and A3 (2). Four papers did not report the shade. In the included studies, specimens’ thickness tested was variable, with the four most frequent thicknesses used being 1 mm (23), 0.5 mm (8), 1.5 mm (6), and 2 mm (1). Concerning measuring instruments, spectrophotometers were used to obtain color coordinates or reflectance values in all the included papers. Among the possible methods to evaluate translucency, the translucency parameter (TP) was the most used method (n = 30), followed by contrast ratio (CR) (n = 11). In only 8 out of 33 papers, the two methods were both used. The data selection process is reported in Figure 1, while the collected information is summarized in Table 2 [11,13,15,20,21,22,23,24,25,26,27,28,29,30,31,32,33,34,35,36,37,38,39,40,41,42,43,44,45,46,47,48,49,50].

## 4. Discussion

Regarding the optical appearance of dental restorations, a tooth-like translucency is required for the fabrication of aesthetically pleasant restorations [47,51,52]. Several optical properties concur in the optical aspect of restoration, such as color attributes (lightness, yellowness/blueness, and greenness/redness), geometric attributes (opacity, transparency, and translucency), and optical phenomena (opalescence, fluorescence, metamerism, gloss, and others). For the present review, the authors focused on translucency, which occurs when a light beam, passing through a material, is partly scattered, reflected, or transmitted. The greater the quantity of light that passes through the material, the higher the translucency [53,54].

Translucency is usually measured with a contrast ratio (CR) [24,51,55,56] or translucency parameter (TP) [14,18,22,24,57,58]. CR is defined as the ratio of the reflectance of a specimen placed over a black backing to that over a white one of known reflectance, and it is defined as an estimate of opacity [59]. CR value ranges from 0 to 1, with 0 corresponding to total translucency (transparency) and 1 corresponding to total opacity. The TP is a color difference (ΔE) between a material measured over white and black backings [18]. Few papers [24,30,42,60] compared TP and CR methods and described possible correlations. Despite these comparative studies, there is no consensus or standard on the method of choice to quantify the translucency of aesthetic restorative materials. Although the usage of TP for translucency measurements is controversial and indicated for ceramic materials with a % of total transmission of at least 50% [60], it is nonetheless considered a well-established test often used in the dental scientific literature [61]. In this review, 30 papers out of 33 used TP, while 11 used CR, with only 8 papers measuring both.

LSGC presents differences in composition that can affect mechanical and optical properties. Variability in the crystalline content may provide significant differences in strength and optical properties [52,61].

In the present review, an electronic search of the literature was performed to retrieve data on the translucency of contemporary LSGC: IPS e.max CAD, Celtra Duo, Suprinity PC, Initial LiSi Block, Amber Mill, N!ce, Obsidian, and CEREC Tessera. As a possible limitation of the study, there is a chance that materials could be on the market with limited territorial availability, depending on market strategies, making it difficult to identify all the materials available worldwide. Also linked to this aspect, the conventional use of text only in English might preclude the full retrieval of information.

Among the examined papers, data for only three of the materials were retrieved: IPS e.max CAD was investigated in all 33 papers, whereas Suprinity PC was investigated in 8, and only 1 study investigated Celtra Duo. According to the classification proposed by Lubauer et al. [16], IPS e.max CAD is a lithium disilicate (crystalline phase in volume/glass phase in volume: 70.3%/29.7%). Suprinity PC is a lithium (di)silicate zirconia-reinforced glass ceramic (ZrO_2_ = 8–12%) (ZLS) with densely distributed round-shaped crystals. The ratio between the crystalline phase and glass phase in volume is 57.1%:42.9%. Like Suprinity PC, Celtra Duo is also a lithium (di)silicate ZLS. The Celtra Duo glass matrix contains 10% zirconia with smaller silicate crystals, and the ratio between the crystalline phase and glass phase in volume is 51%:49%. Data on the translucency of N!ce, Lisi CAD, Tessera, and Amber Mill were not available at the time the literature search was performed.

For CAD/CAM blocks, manufacturers offer formulations with a higher degree of translucency (HT) and formulations with a lower translucency (LT or T). HT blocks are generally characterized by a small number of larger crystals with respect to LT (called ‘T’ for Suprinity PC) that present a larger number of smaller crystals [13]. As a consequence, in low-translucency blocks, there is a higher refractive index of light between crystals and the glass matrix. This effect can cause an increased quantity of light scattering at the crystals–matrix interface, resulting in a higher opacity [13]. The translucency of dental ceramics depends also on other aspects, such as chemical composition, crystal size and morphology [47,51,52], the number of firing cycles [62,63,64,65,66], hydro-thermal aging [67], and thickness [15,47,60,61,62,63,64,65,66,67,68,69,70,71]. Particularly, dimension and shape and the percentage of crystal fraction play a significant role. Several crystals are involved in the process, ranging from lithium silicate and lithium disilicate to lithium aluminosilicate, and others, and from a needle shape of about a few microns prevalent for IPS e.max CAD to a more spheroidal shape of about 2 microns prevalent for Suprinity PC and Celtra Duo to a submicron crystal dimension prevalent for N!ce and Tessera [16]. However, it has to be considered that most of the materials show different crystals simultaneously present in various ratios, making the correlation between a crystal phase and optical behavior quite complex. In this regard, fractal dimension analysis has been proposed to help analyze the materials’ structure [72,73].

Among the materials examined, Amber Mill has a noticeable peculiarity, which is that the translucency can be controlled by firing temperature [74,75]. According to Lubauer et al. [16], Amber Mill has a submicrometric lithium disilicate phase, a lithium silicate phase, and a 6% quartz phase in the nanometric range.

As a probable result of the absence of ISO standards for the measurement of translucency in dentistry, the papers examined in the present review considered different thicknesses, even if most of them (23 out of 33) evaluated translucency with specimens of 1 mm thickness. As LSGC can be used in different thicknesses, ranging from very thin layers for veneers up to thicker ones for crowns, knowledge of the optical behavior of different thicknesses could be clinically relevant. As a commonly reported and quite obvious observation, at clinical thicknesses, the lower the thickness, the higher the translucency.

Unfortunately, only 7 out of 33 papers reported more than one thickness. Vichi et al. [47] reported for IPS e.max CAD A2 LT a CR value of 0.56. Similar values for the same material and same thickness were reported by other papers: Ziyad et al. [50] reported CR = 0.592, Della Bona et al. [30] CR = 0.62, and Nogueira et al. [42] CR = 0.63. Different results were obtained only by Sen and Isler [44], who reported a CR value at 1.00 mm thickness of 0.35. Concerning TP, the values for this material (e.max LT) were in quite a narrow range, between 16 and 18 [15,25,30,34,42], with only a few articles reporting different values: Kwon et al. 12.64 [38], Basso et al. 37.3 [26], and Oh et al. 9.74 [43].

Some of the investigated papers also tested the HT version of IPS e.max CAD. Translucency values were very consistent for several papers, in a range of 18–19 [25,30,36,37,42,48], and CR was in the 0.48–0.58 range [30,42,47]. As for LT, for HT also, few papers reported extremely different TP values, such as Kwon et al. reporting 12.64 [38] and Basso et al. reporting 37.3 [26].

As translucency is a function of thickness, 0.5 mm specimens’ values were highly different from those of 1 mm and less consistent: Skyllouriotis et al. [13] reported, in fact, CR = 0.12 and TP = 52.85 for 0.5 mm IPS e.max CAD HT A2, while Wang et al. [48] reported a TP value of 24 for the same material. Despite that, only 6 papers out of 33 measured thickness values of 1.5 mm or higher for TP, and only 2 papers for CR; it appears evident that translucency decreases as thicknesses increase.

In the present review, eight papers analyzed differences in translucency between IPS e.max CAD and Suprinity PC. Of the papers, 50% [11,21,29,46] reported higher translucency values for IPS e.max CAD, while the other 50% [15,28,32,36] reported higher values for Suprinity PC. These conflicting results were reported even in papers comparing the same combination of nominal translucency (HT, LT, or other) and thickness between the two materials and at different thicknesses (0.5, 1, 1.2, 1.5, and 2 mm). Differences should, therefore, again be linked mainly to the measuring method, such as the type of measuring instrument, backgrounds, and specimen manufacturing procedures, including crystallization processes and surface treatments.

In the only paper investigating Celtra Duo, Alayad et al. [21] compared this material with IPS e.max CAD and Suprinity PC. The latter presented the highest translucency values, followed by IPS e.max CAD and Celtra Duo, which presented similar values. Unfortunately, the authors did not report the shade and translucency degree of the materials used in their study.

As a general remark, the correlation between material nominal translucency (LT, HT) and thickness is a quite complex subject not extensively investigated and with uncertain measurement methodology. These values are clinically relevant, especially when compared to natural tissue values, which are likewise scarcely investigated. The classical study of Yu et al. [12] reported TP values of 18.7 and 16.4 for human enamel and dentin, respectively, measured at 1 mm thickness, and Dietschi et al. [76] reported 0.45 and 0.65 CR values for enamel and dentin, respectively, again measured at 1 mm; they are, in fact, the only references available for the translucency of natural tissues. Moreover, the acceptability and perceptibility thresholds that are pivotal in optical studies are largely investigated for color [77] but scarcely for translucency [78]. Further studies are necessary for a better understanding of the optical behavior of natural tissues and materials regarding translucency at different thicknesses.

## 5. Conclusions

Based on the present review, some conclusions can be drawn:

1. TP is the most used method for evaluating the translucency of LSGC;
2. Most of the studies used a 1 mm specimen thickness;
3. HT and LT (T) formulations were equally investigated; A2 was the most investigated color, followed by A1 and A3;
4. Data are sufficiently available for e.max lithium disilicate but scarce for ZLS VITA Suprinity PC and Celtra Duo and not available for the other searched LDGCs (N!ce, LiSi CAD, Amber Mill, and Tessera);
5. TP was prevalently measured in the range of 16–18 for LT and in the range of 18–19 for HT;
6. CR was prevalently measured in the range of 0.56–0.63 for LT and in the range of 0.48–0.58 for HT.

## Figures and Tables

**Table 1:** Chemical composition of the materials included in the review.

Material	Manufacturer	Definition	Chemical Composition	Thermal Treatment	Translucency Availability
Celtra DUO	Dentsply Sirona, Charlotte, NC, USA	Lithium silicate, zirconia reinforced	58% SiO_2_; 18.5% Li_2_O; 5% P_2_O_5_; 10.1% ZrO; 1.9% Al_2_O_3_; 2% CeO_2_; 1% Tb_4_O_7_	Optional	HT LT
Initial LiSi Block	GC, Tokyo, Japan	Lithium disilicate	55–80% SiO_2_, 10–30% Li_2_O; 5–20% other oxides; pigments: trace **	No	HT LT
N!ce	Straumann, Basel, Switzerland	Lithium fluorosilicate	64–70% SiO_2_; 10.5–12.5% Li_2_O; 0–3% K_2_O; 3–8% P_2_O_5_; 0–0.5% ZrO_2_; 10.5–11.5% Al_2_O_3_; 1–2% CaO; 0–9% pigments; 1–3% Na_2_O	No	HT LT
IPS e.max CAD	Ivoclar Vivadent, Schaan, Liechtenstein	Lithium disilicate	57–80% SiO_2_; 11–19% Li_2_O; 0–13% K_2_O; 0–11% P_2_O_5_; 0–8% ZrO_2_, 0–8% ZnO; 0–12% others + coloring oxides	Yes	HT MT LT MO
Tessera	Dentsply Sirona, Charlotte, NC, USA	Lithium disilicate based	Li_2_Si_2_O_5_: 90% Li_3_PO_4_: 5% Li_0.5_A_l0.5_Si_2.5_O_6_ (LAS, lithium alumino silicate—virgilite): 5%	Yes	HT MT
Suprinity PC	VITA Zahnfabrik, Bad Sackingen, Germany	Lithium silicate, zirconia reinforced	SiO_2_: 56–64% Li_2_O: 15–21% ZrO_2_: 8–12% P_2_O_5_: 3–8% K_2_O: 1–4% Al_2_O_3_: 1–4% CeO_2_: 0–4% pigments: 0–4%	Yes	HT T
Amber Mill	Hass, Gangwon-do, Korea	Lithium disilicate	SiO_2_, Li_2_O, K_2_O, MgO, Al_2_O_3_,P_2_O_5_, other oxides	Yes	HT * MT * LT * MO *

* Different translucencies are obtained through different crystallization processes. ** Company personal communication.

**Table 2:** Included studies and assessed variables.

Article	LSGC Type	Shade	Translucency	Thickness (mm)	TP	CR
Alayad et al. (2021) [21]	IPS e.max CAD	n.r.	n.r.	0.5; 1; 1.5	0.5 mm: 21.33 ± 1.73 1 mm: 13.34 ± 0.59 1.5 mm: 10.28 ± 0.42	
	Suprinity PC	n.r.	n.r.	0.5; 1; 1.5	0.5 mm: 42.49 ± 4.26 1 mm: 42.28 ± 2.79 1.5 mm: 43.25 ± 4.47	
	Celtra Duo	n.r.	n.r.	0.5; 1; 1.5	0.5 mm: 23.94 ± 1.79 1 mm: 14.81 ± 0.53 1.5 mm: 10.39 ± 0.48	
Bagis and Turgut (2013) [22]	IPS e.max CAD	A1	n.r.	0.5	14.49 ± 0.83	
Baldissara et al. (2018) [23]	IPS e.max CAD	A2	LT	1.5		0.84 ± 0.02
Barizon et al. (2013) [24]	IPS e.max CAD	A1	HT	0.7	33.02 ± 0.07	0.25 ± 0.00
Barizon et al. (2014) [25]	IPS e.max CAD	A1	HT	0.7	33.02 ± 0.07	
Basso et al. (2017) [26]	IPS e.max CAD	A1	HT; LT	0.5; 1; 1.5; 2	TP mean (95% confidence interval) values: HT0.7: 44.9 (44.2–45.6) HT1.0: 37.3 (36.8–37.8) HT1.5: 29.4 (28.8–30.0) HT2.0: 22.9 (22.6–23.2) LT0.7: 34.4 (33.9–34.9) LT1.0: 27.3 (26.7–27.9) LT1.5: 22.2 (21.7–22.7) LT2.0: 15.5 (15.2–15.8)	
Brescansin et al. (2021) [27]	IPS e.max CAD	A2	HT	1.5	* Data retrieved from graph: 14.1	
Carrabba et al. (2017) [20]	IPS e.max CAD	n.r.	LT	1		0.56
Campanelli et al. (2021) [28]	IPS e.max CAD	n.r.	n.r.	1.2	29.16–30.32 (depending on firings)	
	Suprinity PC	n.r.	n.r.		26.89–29.17 (depending on firings)	
Caprak et al. (2019) [29]	IPS e.max CAD	A2	HT	2	10.16 ± 0.50	
	Suprinity PC	2M2	HT	2	11.92 ± 0.78	
Della Bona et al. (2014) [30]	IPS e.max CAD	A1; A2; A3	LT; HT	1	LT A1: 16.79 ± 0.35 HT A1: 18.51 ± 0.59 LT A2: 17.35 ± 0.81 HT A2: 18.97 ± 0.16 LT A3: 18.62 ± 1.06 HT A3: 18.98 ± 0.28	LT A1: 0.64 ± 0.01 HT A1: 0.59 ± 0.01 LT A2: 0.62 ± 0.01 HT A2: 0.58 ± 0.01 LT A3: 0.60 ± 0.02 HT A3: 0.57 ± 0.01
Gasparik et al. (2019) [31]	IPS e.max CAD	A3	HT; LT	1	HT 18.87 (0.32) LT 17.79 (0.24)	
Gunal and Ulusoy (2018) [15]	IPS e.max CAD	A2	LT	0.5; 1.0	0.5 mm: 27.51 ± 0.94 1 mm: 16.13 ± 0.33	
	Suprinity PC	A2	T		0.5 mm: 23.30 ± 0.71 1 mm: 14.26 ± 0.52	
Juvantee and Uasuwan (2019) [32]	IPS e.max CAD	A2	n.r.	1.2	Depending on thermal tempering speed: (S)low, (N)ormal, (S)low S:5.53 ± 0.17 N:5.49 ± 0.17 F:5.36 ± 0.06	Depending on thermal tempering speed: (S)low, (N)ormal, (S)low S:0.821 ± 0.006 N: 0.822 ± 0.006 F:0.826 ± 0.002
	Suprinity PC	A2	n.r.		S: 2.44 ± 0.24 N: 4.05 ± 0.3 F: 3.79 ± 0.17	S: 0.958 ± 0.006 N: 0.911 ± 0.010 F: 0.919 ± 0.006
Kanpalta et al. (2022) [33]	IPS e.max CAD	A2	LT	1	18.93 ± 0.52	
Karci and Demir (2019) [34]	IPS e.max CAD	A2	LT	1	18.11 ± 1.46	0.56 ± 0.02
Kulkarni et al. (2020) [35]	IPS e.max CAD	n.r.	n.r.	1	8.49 ± 1.01	
Kurt et al. (2020) [36]	IPS e.max CAD	A2	HT	1	Before crystallization: 18.53 ± 0.64 After crystallization: 18.25 ± 0.41	
	Suprinity PC	A2	HT		Before crystallization: 16.16 ± 0.70 After crystallization: 16.44 ± 0.53	
Kurtulmus-Yilmaz et al. (2019) [37]	IPS e.max CAD	A2	HT	1	20.1 ± 0.5	
Kwon et al. (2018) [38]	IPS e.max CAD	A1	HT; LT	1	HT: 12.64 ± 0.48 LT: 9.28 ± 0.36	
Lawson and Maharishi (2020) [39]	IPS e.max CAD	BL1	LT	1	9.33 ± 0.56	
Ledić et al. (2015) [40]	IPS e.max CAD	A2; C2; B3	HT	0.8	A2: 14.17 ± 0.85 C2: 13.87 ± 1.17 B3: 14.27 ± 0.50	
Monaco et al. (2020) [41]	IPS e.max CAD	A2	LT	2	14.54 ± 1.64	
Nogueira and Della Bona (2013) [42]	IPS e.max CAD	A2	LT; HT	1	LT: 17.3 ± 0.81 HT: 19.0 ± 0.16	LT: 0.63 ± 0.02 HT: 0.58 ± 0.00
Oh et al. (2018) [43]	IPS e.max CAD	A2	HT;LT	0.5; 1; 2; 4	HT: 0.5 mm: 20.79 ± 0.10 1 mm: 13.39 ± 0.04 2 mm: 5.27 ± 0.18 4 mm: 1.21 ± 0.20 LT: 0.5 mm: 17.64 ± 0.05 1 mm: 9.74 ± 0.10 2 mm: 1.93 ± 0.03 4 mm: 0.58 ± 0.10	
Şen and Us (2017) [44]	IPS e.max CAD	A2	HT	1.2	26.0 ± 0.6	
	Suprinity PC	2M2	HT		31.0 ± 1.0	
Sen and Isler (2020) [44]	IPS e.max CAD	A1	LT	0.5; 1; 1.5	* Data retrieved from graph: 0.5 mm: 32 1 mm: 25 1.5 mm: 18	* Data retrieved from graph: 0.5 mm: 0.28 1 mm: 0.34 1.5 mm: 0.53
Skyllouriotis et al. (2017) [13]	IPS e.max CAD	A2	LT; HT	0.5	LT: 40.24 HT: 52.85	LT: 0.25 HT: 0.17
Supornpun et al. (2021) [45]	IPS e.max CAD	A2	HT	1; 1.25; 1.5; 1.75; 2	1 mm: 12.44 ± 0.06 1.25 mm: 11.24 ± 0.53 1.5 mm: 9.72 ± 0.08 1.75 mm: 8.67 ± 0.02 2.00 mm: 7.85 ± 0.26	
Turgut et al. (2019) [46]	IPS e.max CAD	A1	LT	1	20.6 ± 0.5	
	Suprinity PC	A1	T		22.5 ± 0.7	
Vichi et al. (2014) [47]	IPS e.max CAD	A2	LT; HT; MO	0.5; 1.0		HT 0.5 mm: 0.35 ± 0.01 HT 1.0 mm: 0.48 ± 0.01 LT 0.5 mm: 0.43 ± 0.02 LT 1.0 mm: 0.56 ± 0.02 MO 0.5 mm: 0.50 ± 0.03 MO 1.0 mm: 0.71 ± 0.02
Wang et al. (2020) [48]	IPS e.max CAD	A2	HT; MO	0.5; 1	* Data retrieved from graph: 0.5 mm: 24 1.0 mm: 17	
Ziyad et al. (2021) [50]	IPS e.max CAD	A2	LT	1	20.439 ± 0.86	0.592 ± 0.016

n.r. = not reported; * Data retrieved from graph; LT = Low Translucency; T = Translucent; HT = High Translucency; MO = Medium Opacity.

## Data Availability

The data presented in this study are available on request from the corresponding author. The data are not publicly available due to the university’s policy on access.
